# N6-Adenosine Methylation of miRNA-200b-3p Influences Its Functionality and Is a Theranostic Tool

**DOI:** 10.1016/j.omtn.2020.08.010

**Published:** 2020-08-14

**Authors:** Joséphine Briand, Aurélien A. Sérandour, Arulraj Nadaradjane, Gwenola Bougras-Cartron, Dominique Heymann, Benjamin Ory, François M. Vallette, Pierre-François Cartron

**Affiliations:** 1 CRCINA, INSERM, Université de Nantes, Nantes, France; 2 Equipe Apoptose et Progression Tumorale, LaBCT, Institut de Cancérologie de l’Ouest, Saint Herblain, France; 3 Cancéropole Grand-Ouest, Réseau Niches et Epigénétique des Tumeurs (NET), Nantes, France; 4 EpiSAVMEN Network (Région Pays de la Loire), Nantes, France; 5 LabEX IGO, Université de Nantes, Nantes, France; 6 Ecole Centrale Nantes, Nantes, France; 7 INSERM, U1238, Université de Nantes, Nantes, France

**Keywords:** miRNA methylation, glioblastoma, adenosine methylation, prodrug, biomarker

## Abstract

MicroRNAs (miRNAs or miRs) play crucial roles in biological and pathological processes. Some miRNAs also appear as promising biomarkers and therapeutic tools. However, the epitranscriptomic regulation of miRNAs is not yet fully elucidated in all of their fields of application. We report that adenosine methylation of miR-200b-3p inhibits its repressive function toward its mRNA targets such as XIAP by blocking the formation of the miRNA/3′ UTR^mRNA^ duplex. Our data indicate that the adenosine methylation of miR-200b-3p is associated with the survival of glioblastoma patients. Collectively, our data support the idea that the adenosine methylation of miR-200b-3p can be used as a prodrug having a selective cytotoxicity against cancer cells (while being harmless to peripheral blood mononuclear cells [PBMCs], astrocytes, neurons, and hepatocytes).

## Graphical Abstract

**Figure:**
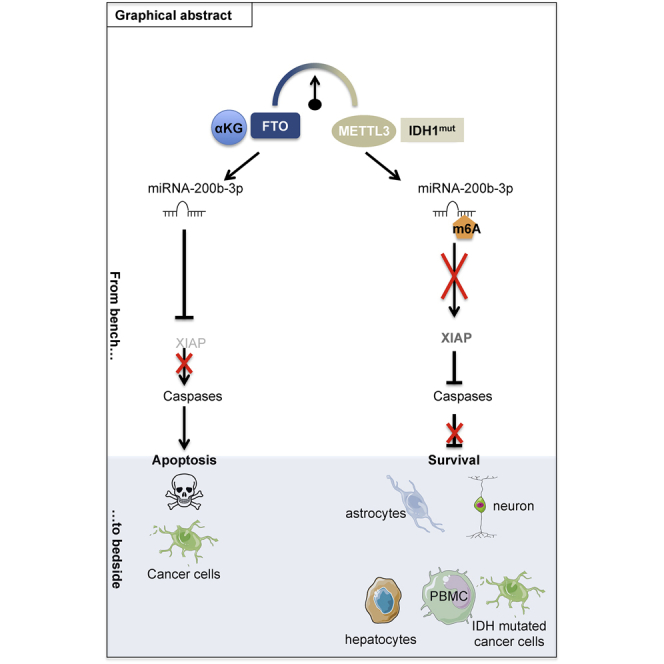

## Teaser

Through the study of miR-200b-3b, Cartron and colleagues report two innovative points in the epitranscriptomics field: adenosine methylation of miRNA can abrogate the repressive miRNA function and can be used as a prodrug form of miRNA in order to limit its off-target action.

## Introduction

MicroRNAs (miRNAs or miRs) are short non-coding RNAs (ncRNAs) that regulate protein expression toward their function as translational repressors. Thus, miRNAs are crucial regulators of many cellular processes, including proliferation, apoptosis, immunogenicity, development, and differentiation. miRNA biogenesis can be epigenetically regulated in both physiological and pathological conditions toward the DNA methylation of miRNA genes. Wang et al.^1^ reported that the expression of approximately 50% of miRNA genes is putatively regulated by DNA methylation since they are associated with CpG islands. A variety of DNA methylation-specific methyl-CpG-binding domain (MBD) proteins were also found to transcriptionally regulate miRNA genes.^2^ Finally, Malumbres et al.^3^ also reported that the expression of miRNA genes is also regulated through histone modifications, such as lysine methylation and acetylation.

Several publications have reported that chemical modifications can occur in miRNA and that these modifications regulate the miRNA processing or functionality. Among these modifications, some can affect the phosphate at the 5′ end of the miRNA. Thus, Xhemalce et al.^4^ reported that the BCDIN3D-mediated phospho-dimethylation of miRNAs (such as precursor [pre-]miR-145) negatively regulates miRNA maturation and impacts the tumorigenic phenotype. Other chemical modifications of miRNA affect the internal bases of miRNA. Alarcón et al.^5^ and Berulava et al.^6^ reported that miRNA can be adenosine methylated and that the presence of this methylation promotes the initiation of miRNA biogenesis and increases the stability of adenosine-methylated miRNAs, respectively. Konno et al.^7^ also reported that miRNA can be adenosine methylated. Additionally, in their report, the authors introduced the idea that the adenosine methylation of miRNA can be used as a biomarker for the diagnosis of early-stage cancer. Pandolfini et al.^8^ reported that miRNA can be guanosine methylated and that this methylation inhibits the miRNA maturation. Recently, our laboratory published that miRNAs can be cytosine methylated and that the presence of this methylation represses the miRNA function.^9^

Several enzymes catalyze these base modifications, including METTL1 (methyltransferase-like protein 1, UniProt: Q9UBP6) and DNMT3A (DNA [cytosine-5]-methyltransferase 3A, UniProt: Q9Y6K1), which promote the guanosine and cytosine methylation of miRNAs, respectively.^8^^,^^9^ The complex methyltransferase-like 3 (METTL3)-Wilms’ tumor 1-associating protein (WTAP)- methyltransferase-like 14 (METTL14) is described as a miRNA adenosine methylase or writer, while FTO (fat mass and obesity-associated protein, UniProt: Q9C0B1) and ALKBH5 (alkylated DNA repair protein alkB homolog 5, UniProt: Q6P6C2) are described as miRNA adenosine demethylases or erasers.^5^^,^^6^^,^^10^, ^11^, ^12^, ^13^ Interestingly, these two enzymes are α-ketoglutarate (αKG)-dependent, suggesting that the adenosine methylation of miRNA can be regulated by the intracellular level of αKG. αKG is a Krebs cycle metabolite. It is formed from isocitrate by oxidative decarboxylation catalyzed by IDH (isocitrate dehydrogenase) proteins and plays a key role in multiple metabolic and cellular pathways via its co-substrate role of several enzymes such as FTO and ALKBH5.^14^ Thus, in theory, a high level of αKG should increase the FTO activity and should promote a decrease of adenosine methylation of miRNA.

Despite these undeniable advances, further studies of the molecular mechanisms governing the chemical modifications of miRNA in a tumor context are required in order to increase the understanding of the role played by these modifications in tumors.

We focused our study on the impact of presence of N6-adenosine methylation in miRNA-200b-3p in samples of patients suffering from glioblastoma multiforme (GBM). Our study is particularly focused on the impact of miRNA-200b-3p and its adenosine methylation on the expression of XIAP (X-linked inhibitor of apoptosis protein, UniProt: P98170). XIAP acts as an anti-apoptotic protein via the inhibition of caspase-3 and caspase-7 activation, and high XIAP expression is associated with poor survival in several solid tumors.^15^^,^^16^ Thus, the miR-200b-3p-mediated repression of XIAP mRNA expression appears as a mechanism governing the caspase-3 and caspase-7 activity and apoptosis. In theory, in the presence of miR-200b-3p, XIAP mRNA expression is repressed and caspase-3 and caspase-7 can be activated to promote apoptosis. However, in the absence or inactivation of miR-200b-3p, XIAP is expressed, blocks caspase-3 and caspase-7 activation, and therefore inhibits apoptosis. This is why it is important to combine miR-200b-3p expression study with its action capacity.

## Results

### The m6A Methyltransferase METTL3, the m6A Demethylase FTO, and αKG Regulate the N6-Adenosine Methylation of miR-200b-3p

**Figure 1:**
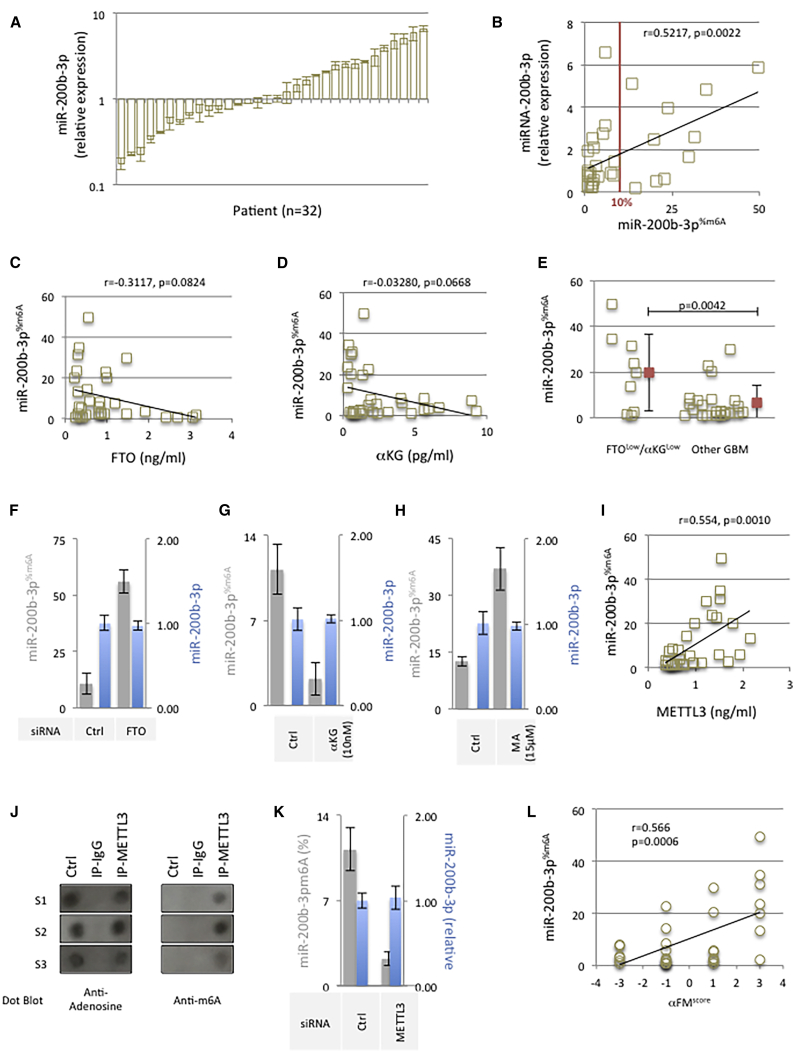
The m6A Methyltransferase METTL3, the m6A Demethylase FTO, and α-Ketoglutarate Regulate the N6-Adenosine Methylation of miR-200b-3p (A) Each bar represents the relative expression level of miR-200b-3p (miR-200b-3p^exp^) in 32 samples of GBM. After miRNA extraction from tumors, qRT-PCR was performed to evaluate the relative expression level of miRNA-200b-3p by using non-tumor brain samples as a reference and SNORD6.1 as a housekeeping miRNA. (B) Correlation between the relative expression level of miR-200b-3p and the percentage of N6-adenosine methylation in miR-200b-3p (miR200b-3p^%m6A^). This percentage was calculated via the realization of RNA immunoprecipitation (IP) performed with an anti-m6A antibody followed by qPCR analysis (miRIP^m6A^-qPCR) (as previously described by Berulava et al.^6^). Each square represents a GBM patient. (C) Absence of correlation between the relative expression level of FTO and the percentage of N6-adenosine methylation in miR-200b-3b. A human FTO ELISA kit (Tebu-Bio, France) was used to estimate the FTO expression. Each square represents a GBM patient. (D) Absence of correlation between the relative presence of α-ketoglutarate (αKG) and the percentage of N6-adenosine methylation in miR-200b-3p. An αKG assay kit (Abcam, France) was used to estimate the relative presence of αKG. Each square represents a GBM patient. (E) High percentage of N6-adenosine methylation in miR-200b-3p is observed in GBM harboring a low level of FTO and αKG. Each square represents a GBM patient. Red squares represent the average ± standard deviation of the two considered subgroups. (F) Impact of the downregulation of FTO on the adenosine methylation percentage of miR-200b-3p%^m6A^ calculated through the realization of miRIP^m6A^-qPCR as previously described. (G) Impact of the αKG treatment on the adenosine methylation percentage of miR-200b-3p (miRNA-200b-3p^%m6A^). (H) Impact of the meclofenamic acid (MA) treatment on miRNA-200b-3p^%m6A^ in U87 cells. After MA treatment (15 μM/24 h, Santa Cruz, France), the miRNA-200b-3p^%m6A^ was calculated through the realization of miRIP^m6A^-qPCR as previously described. (I) Correlation between the relative expression level of METTL3 and the percentage of N6-adenosine methylation in miR-200b-3p. Each square represents a GBM patient. (J) Dot blot illustrating the presence of adenosine methylation in mimetic miR-200b-3p in the presence of METTL3 IP product. Adenosine detection is used as a control. S1/S2/S3 are three independent experiments. Ctrl, synthetic miR-200b-3p adenosine unmethylated; IP-IgG, product of IP performed with anti-IgG (the absence of an adenosine signal indicates that miRNA was not unspecifically immunoprecipitated); IP-METTL3, product of IP performed with anti-IgG. Products of IP were obtained in non-denaturing conditions in order to conserve enzymatic activity according to the manufacturer’s indications. (K) METTL3 knockdown (by siRNA approach) decreases the percentage of adenosine methylation of miR-200b-3p. (L) αFM^score^ reflects the METTL3, FTO, and αKG expression levels in the 32 GBM samples. Each circle represents a GBM patient. The αFM^score^ is higher when the percentage of adenosine methylation of miR-200b-3p is higher.

Studies have reported that miR-200 and particularly miR-200b-3p play a role in GBM.^17^, ^18^, ^19^, ^20^ Berulava et al.^6^ have identified the presence of N6-methyladenosine (m6A) in certain miRNAs such as miR-200b-3p. In agreement with these findings, we have investigated the miR-200b-3p level expression (miR-200b-3p^exp^) and the percentage of miRNA-200b-3p containing m6A (miR-200b-3p^%m6A^) in a collection of 32 GBM samples (Table S1). qRT-PCR experiments indicated a high level of heterogeneity in miR-200b-3p^exp^ with a maximum/minimum ratio equal to 37.6 (Figure 1A). RNA immunoprecipitation (RIP) performed with an anti-m6A antibody followed by qPCR analysis (miRIP^m6A^-qPCR) indicated that 10 out of 32 tumors contained a miR-200b-3p^%m6A^ >10% (Figure 1B). In addition, we observed a correlation between miR-200b-3p^%m6A^ and miR-200b-3p^exp^ (p = 0.0022) (Figure 1B).

In order to identify the molecular mechanisms governing the N6-adenosine methylation of miR-200b-3p in GBM patients, we first focused our analyses on FTO and αKG, since FTO is an adenosine demethylase that requires αKG to catalyze the adenosine demethylation.^11^ In our collection of 32 GBMs, Pearson’s correlation tests showed an absence of significant correlation of FTO expression level with miR-200b-3p^%m6A^ (p = 0.0824) (Figure 1C) and between αKG and miR-200b-3p^%m6A^ (p = 0.0668) (Figure 1D). To consider these two parameters, we isolated GBM samples harboring a low FTO expression level (lower than median) and a low αKG level (lower than median) (FTO^low^/αKG^low^) from the other GBM samples (Figure S1). Based on this subdivision, we noted that GBM samples harboring FTO^low^/αKG^low^ were more m6A methylated than other GBM samples (p = 0.0042) (Figure 1E). Thus, we conclude that both FTO and αKG affect the m6A methylation level of miR-200b-3p; that is, the N6-adenosine methylation level of miR-200b-3p is elevated when FTO and αKG levels are lower. The involvement of FTO and αKG in the N6-adenosine methylation of miRNA was also supported by the fact that small interfering RNA (siRNA) directed against FTO increased miR-200b-3p^%m6A^ (Figure 1F; Figure S2), αKG treatment decreased miR-200b-3p^%m6A^ (Figure 1G), and meclofenamic acid (MA, a selective FTO inhibitor^21^) increased the miR-200b-3p^%m6A^ (Figure 1H). In addition, we noted that the knockdown of ALKBH5 (an RNA adenosine demethylase^10^) did not change the miR-200b-3p^%m6A^ (Figure S3). Thus, all of these results support the idea that FTO and αKG act in concert to decrease the adenosine methylation of miR-200b-3p.

Based in part on Alarcón et al.^5^ having identified that METTL3 methylates primary (pri-)miRNA in mammalian cells, we hypothesized that METTL3 could be implicated in the adenosine methylation of miR-200b-3p. To support this hypothesis, we first observed a significant correlation between miR-200b-3p^%m6A^ and the METTL3 expression level (p = 0.0010) (Figure 1I). Second, acellular experiments indicated that the immunoprecipitate of METTL3 (i.e., METTL3-including complexes) methylates miRNA-200b-3p *in vitro* (Figure 1J). Third, METTL3 knockdown (siRNA method) decreased the level of m6A in miR-200b-3p (Figure 1K; Figure S4). To conclude, these three distinct experiments implicate METTL3 as a writer of N6-adenosine methylation of miR-200b-3p.

All of the above results suggest that αKG, FTO, and METTL3 collectively influence the presence of m6A in miR-200b-3p. In order to take into consideration the influence of these three parameters on the level of adenosine methylation of miR-200c-3p, we have calculated what we call the αFM^score^. For each GBM sample, +1 is applied when the expression of αKG, FTO, and METTL3 is predicted to increase the N6-adenosine methylation, i.e., when the αKG and FTO expressions are lower or equal to the median value of our cohort and when METTL3 expression is higher than the median value of our cohort; −1 is applied when the expression of αKG, FTO, and METTL3 is predicted to decrease the N6-adenosine demethylation, i.e., when the αKG and FTO expressions are higher than the median value of our cohort and when METTL3 expression is lower or equal to the median value of our cohort. For example, a GBM harboring a high level of αKG and FTO and a low level of METTL3 has an αFM^score^ equal to +1, while another GBM harboring a low level of αKG and FTO and a low level of METTL3 has an αFM^score^ equal to +3. Thus, we noted that the αFM^score^ and the percentage of presence of m6A in miR-200b-3p were significantly correlated in our collection of 32 GBM samples (p = 0.0006) (Figure 1L).

Taken together, our data support the idea that METTL3, FTO, and αKG are involved in the regulation of the N6-adenosine methylation of miR-200b-3p.

### The N6-Adenosine Methylation of miR-200b-3p Limits Its Translational Repressor Function toward Anti-apoptotic Players and Confers Poor Prognosis in GBM Patients

With XIAP^mRNA^ being identified as a target of miR-200b-3p (according to the miRTarBase website), we next investigated whether there is a link between miR-200b-3p^exp^, miR-200b-3p^%m6A^, and the XIAP expression in our collection of 32 GBM samples.

**Figure 2:**
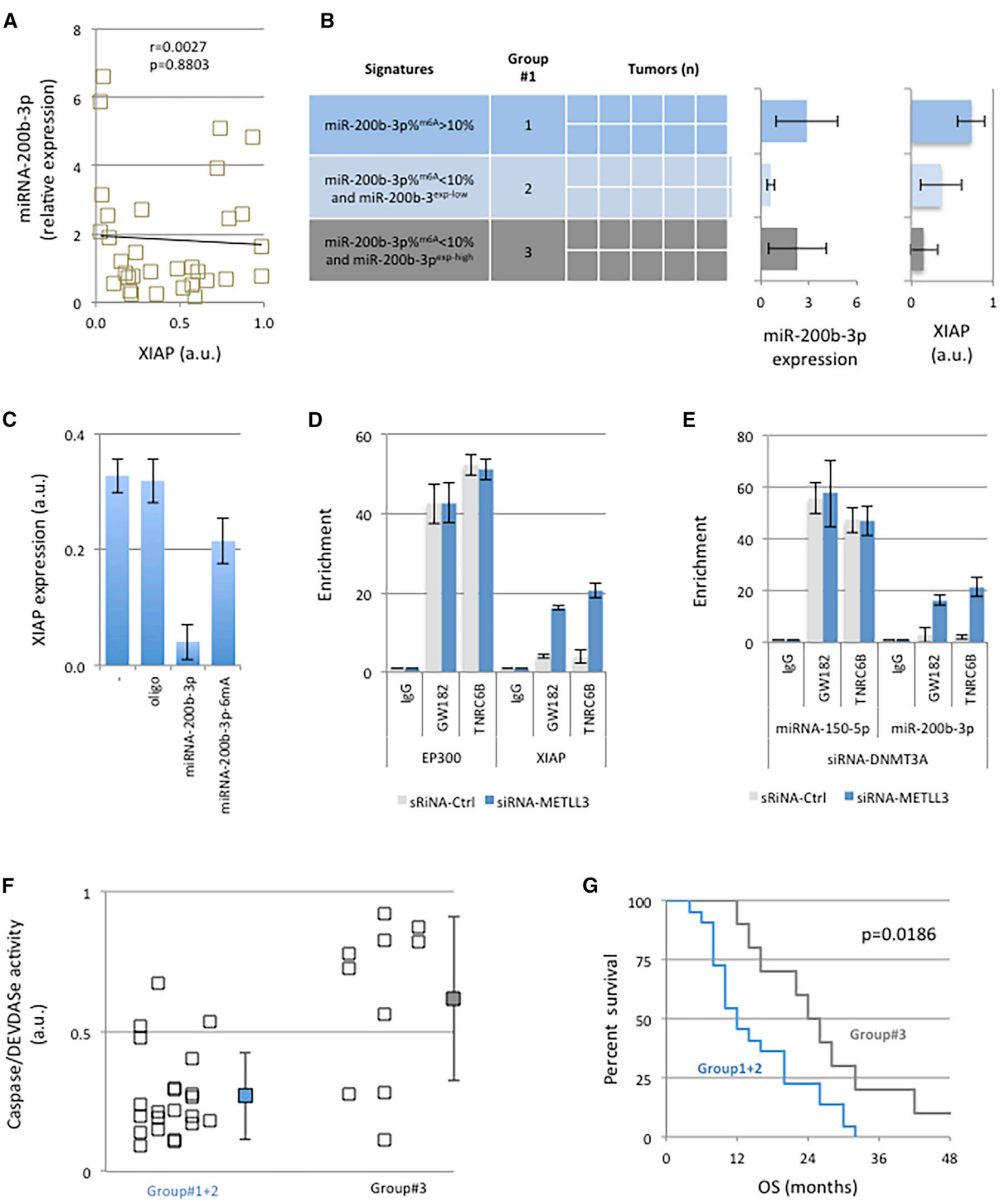
The N6-Adenosine Methylation of miR-200b-3p Limits Its Translational Repressor Function toward Anti-apoptotic Players and Confers Poor Prognosis in GBM Patients (A) Absence of correlation between the relative expression level of XIAP and miRNA-200b-3b (miR200b-3p^exp^) in all 32 patients included in our study. A human XIAP ELISA kit (Abcam, France) was used to estimate the relative expression level of XIAP. Each square represents a GBM patient. (B) Samples were stratified according to the miR-200b-3p^exp^ and miR-200b-3p%^m6A^ parameters in order to distinguish the three indicated groups. Each box represents a sample/patient. For each group, the average of the XIAP expression was analyzed with a human XIAP ELISA kit (Abcam, France) and is represented on the graph. (C) Impact of the *in vitro* N6-adenosine methylation of miRNA-200b-3p^mimetic^ on the expression of the XIAP (human XIAP ELISA kit, Abcam, France). (D) Cross-linking immunoprecipitation and qPCR (CLIP-qPCR) were used to investigate the 3′ UTR/XIAP and 3′ UTR/EP300 (internal control) enrichments on GW182, TNRC6B, and IgG (negative control). Experiments were performed using the RiboCluster Profiler kit (CliniScience, France) according to the manufacturer’s instructions. (E) CLIP-qPCR was used to investigate the miR-150-5p (internal control) and miR-200b-3p enrichments on GW182, TNRC6B, and IgG (negative control). Experiments were performed using the RiboCluster Profiler kit (CliniScience, France) according to the manufacturer’s instructions. (F) For each sample, DEVDase activity was estimated as previously described.^22^ Each open square represents a sample. A blue square represents the average of samples having miR-200b-3p^m6A^ >10% or miRNA-200b-3p^exp-low^. A gray square represents the average of samples having miR-200b-3p^m6A^ <10% or miRNA-200b-3p^exp-high^. (G) Kaplan-Meier representation of survival curves for GBM patients whose tumors are characterized by a miR-200b-3p^m6A^ >10% or a miRNA-200b-3p^exp-low^ (in blue) and by a miR-200b-3p^m6A^ <10% and a miRNA-200b-3p^exp-high^ (in gray).

Our study did not correlate miR-200b-3p^exp^ and the XIAP expression when all GBM samples were considered (p = 0.8803) (Figure 2A).

We then extended our study by dividing our samples into three groups by taking into consideration the adenosine methylation percentage of miR-200b-3p (Figure 2B). Group 1 included samples with miR-200b-3p^%m6A^ >10%. Group 2 included samples with a percentage of miR-200b-3p^%m6A^ <10% and miR-200b-3p^exp^ inferior to the median (miR-200b-3p^exp-low^). Group 3 included samples with miR-200b-3p^%m6A^ <10% and an expression level of miR-200b-3p superior to the median (miR-200b-3p^exp-high^).

For all samples having miR-200b-3p^%m6A^ <10% (groups 2 and 3), we noted that XIAP expression is inversely correlated with miR-200b-3p^exp^ (Figures 2B; Figure S5). These data are consistent with the dogma saying that miRNA is a post-transcriptional repressor.

Surprisingly, we noted that the average of XIAP expression of group 1’s samples was higher than the ones of the two other groups (Figure 2B). These results suggest that miR-200b-3p regulates XIAP expression when its sequence does not contain m6A (or a level inferior to 10%) and that the m6A presence in miR-200b-3p could abrogate the post-transcriptional repressor function of this miRNA.

To investigate this hypothesis, U251 cells were treated with an unspecific oligonucleotide (negative control), miR-200b-3p^mimetic^, or m6A-modified miR-200b-3p^mimetic^. As expected, we did not observe any change in XIAP expression when cells were treated with an unspecific oligonucleotide, while XIAP expression strongly decreased when cells were treated with miR-200b-3p^mimetic^ (Figure 2C). Interestingly, we noted that this decrease was less efficient when cells were treated with the same quantity of m6A-modified miR-200b-3p^mimetic^ (Figure 2C). Thus, it appears that the presence of m6A in miR-200b-3p abrogates the post-transcriptional repressor function of this miRNA toward XIAP^mRNA^.

We next performed cross-linking immunoprecipitation (IP) and qPCR (CLIP-qPCR) analyses to determine whether the adenosine methylation of miR-200b-3p influences the endogenous formation of the 3′ UTR-mRNA-XIAP/miR-200b-3p duplex. In our assays, IP was performed via an antibody directed against GW182 and TNRC6B (i.e., two proteins of the RNA-induced silencing complex [RISC] complex having a central role in miRNA-mediated silencing), and qPCRs were performed to detect the enrichment/presence of miRNA and 3′ UTR^mRNA^ on the GW182- and TNRC6B-mediated coIP products. CLIP-qPCRs were performed from samples with knockdown of METTL3 in order to estimate the impact of the loss of adenosine methylation on the GW182- and TNRC6B-mediated coIP of miRNAs and mRNAs. The miR-150-5p/3′ UTR-mRNA-EP300 duplex was considered as a control. The choice of this control was dictated by the fact that miR-150-5p is not adenosine methylated and the fact that miR-150-5p targets 3′ UTR-mRNA-EP300.

We first noted that miR-150-5p and 3′ UTR-mRNA-EP300 were present in GW182- and TNRC6B- mediated coIP products, and this was independent of the METTL3 knockdown (Figures 2D and 2E). Second, we noted that the METTL3 knockdown increased the presence of miR-200b-3p and 3′ UTR-XIAP in the GW182 and TNRC6B immunoprecipitates (Figures 2D and 2E). Thus, these last results indicate that the METTL3-mediated adenosine methylation status of miR-200b-3p influences the endogenous formation of the 3′ UTR-mRNA-XIAP/miR-200b-3p duplex.

By affecting the expression of XIAP, an apoptotic player, our data suggest that the expression level and the N6-adenosine methylation level of miR-200b-3p could affect the intrinsic apoptosis level of tumors. To investigate this hypothesis, we analyzed the caspase/DEVDase activity as a marker of the intrinsic apoptosis level of tumors. Our work indicates that tumors harboring the miRNA-200b-3p^exp-low^ signature or the miR-200b-3p^%m6A^ >10% signature have a lower intrinsic apoptosis level (Figure 2F).

Finally, we observed that patients whose tumors harbored the miRNA-200b-3p^exp-low^ signature or the miR-200b-3p^%m6A^ >10% signature have a lower survival outcome than did the other GBM patients (Figure 2G).

### m6A-miR-200b-3p Appears as a Promising Tool in Anti-GBM Therapy

**Figure 3:**
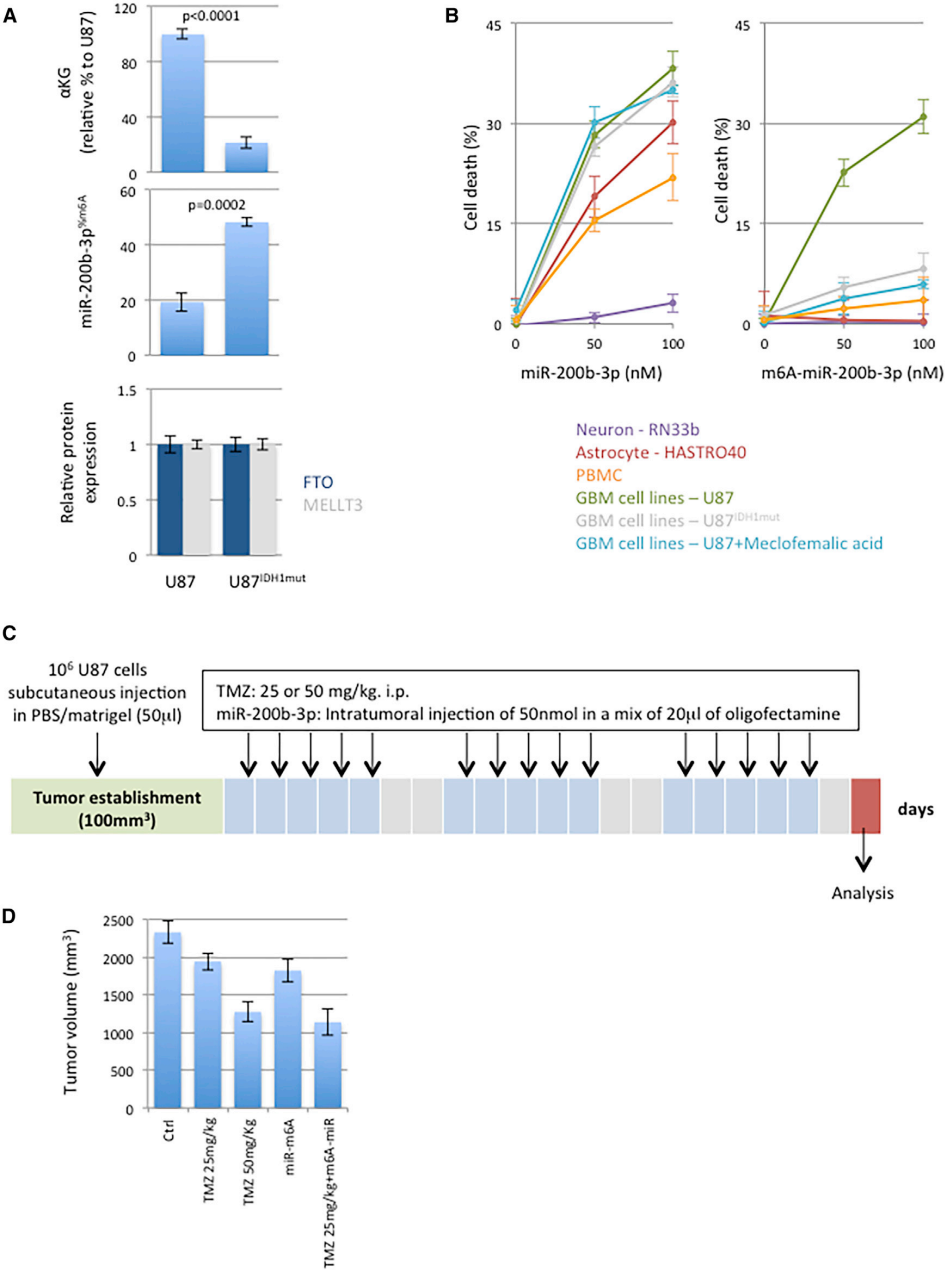
The N6-Adenosine Methylation of miR-200b-3p Selectively Induces Apoptosis in Cancer Cells and Has an Anti-tumor Growth Effect (A) IDH mutation in U87 cells induces a decrease of the αKG rate and an increase of miR-200b-3p^%m6A^ without FTO or METTL3 expression modification. (B) miR-200b-3p promotes cell death by itself in cancerous and non-cancerous cells (excepted neuron RN33b), while miR-200b-3b induces apoptosis by itself in U87 cells only. A lactate dehydrogenase (LDH)-cytotoxicity assay kit (Abcam, France) was used to estimate the cell death 24 h after the m6A-miR-200b-3b incubation. (C) Representation of our *in vivo* model of U87-induced GBM. Mice were xenografted with U87 cells. When tumor volume was equivalent to 100 mm^3^, mice were treated with TMZ and/or miR-200b-3p 5 days per week. Tumor volumes were measured after 3 weeks of treatment. (D) Impact of the adenosine-methylated form of miR-200b-3p on the tumor growth in mice model.

Based on the fact that miR-200b-3p affects the intrinsic apoptosis level, we extended our study by investigating whether miR-200b-3p and m6A-miR-200b-3p could be used as a therapeutic tool. For this purpose, miR-200b-3p- and m6A-miR-200b-3p-induced cell death was measured from a panel of cells representing human brain cells, including astrocytes (HAST40), neurons (RN33b), and astrocytoma (U87). We included this panel of U87^IDH1mut^ cells since IDH1 mutation is observed in GBM. Additionally, we observed that the presence of IDH1 mutation decreased αKG and increased the adenosine methylation of miR-200b-3p in a context of the FTO and METTL3 expression level being unchanged (Figure 3A). Meclofenamic acid was also used as a FTO inhibitor.^21^ Because peripheral blood is the place where exposure to chemicals occurs, PBMCs (peripheral blood mononuclear cells) were also included in our study. First, our data indicated that miRNA-200b-3p induced cell death in all cells with the exception of neurons (RN33b cell line) (Figure 3B). Second, we observed that m6A-miR-200b-3p induced cell death in U87 cells, but not in U87^IDH1mut^, U87^meclofenamic^, PBMCs, neurons, and astrocytes (Figure 3B). In other terms, these data suggest that the ability of m6A-miR-200b-3p to induce cell death occurs in cancer cells and not in non-cancerous cells such as PMBCs, neurons, and astrocytes. Based on our knowledge, the absence of massive m6A-miR-200b-3p-induced cell death in U87^IDH1mut^ could be associated to the fact that these cells have a lower quantity of αKG, i.e., a lower quantity of the enzyme co-factor (FTO) catalyzing the adenosine demethylation of miR-200b-3p. Additionally, the fact that the meclofenamic acid treatment abrogated the m6A-miR-200b-3p-induced cell death in U87 cells confirmed the involvement of FTO in this process (Figure 3B).

We then investigated the putative anti-GBM effect of m6A-miR-200b-3p in an *in vivo* model of GBM. For this purpose, U87-induced GBMs were generated by xenografts in mice. When the volume of the U87-induced GBMs was close to 100 mm^3^, three mice were randomly untreated, treated with temozolomide (TMZ), and/or treated with m6A-miR-200b-3p (Figure 3C). The option to use TMZ is due to the fact that this alkylating agent is the chemotherapeutic agent included in the current standard care protocol in GBM treatment.^23^

By comparing the effect of the TMZ treatment with the effect of the m6A-miR-200b-3p treatment, we could clearly see that the m6A-miR-200b-3p treatment has a similar efficiency than with TMZ (25 mg/kg) treatment (Figure 3D). We also noted that the m6A-miR-200b-3p+TMZ (25 mg/kg) treatment has the same efficiency than with the TMZ (50 mg/kg) treatment (Figure 3D).

### miR-200b-3p Could Also Be Used as a Therapeutic Tool in Other Cancer Types

**Figure 4:**
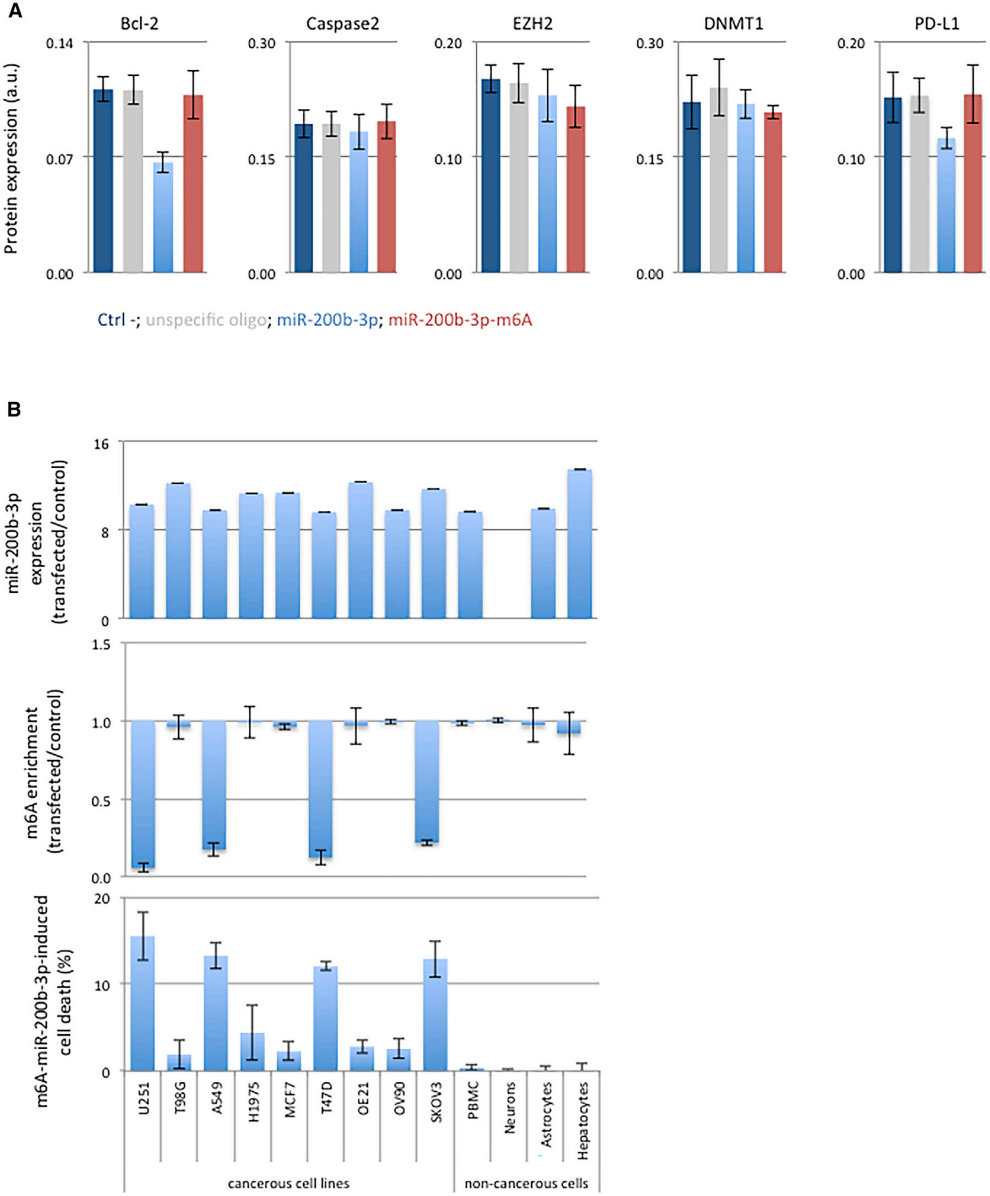
miR-200b-3p Could Also Be Used as a Therapeutic Tool in Other Cancer Types (A) Protein expression of different targets of mir-200b-3p has been studied by ELISA in U87 cells treated with an unspecific oligonucleotide or miR-200b-3p adenosine methylated or not. miR-200b-3p decreases Bcl-2 and PD-L1 expression only when not methylated and has no effect on other proteins studied. (B) In cell lines transfected with m6A-miR-200b-3p, cell death was induced in several cancer cell line types, when cell lines were able to demethylate this miRNA.

The above data are focused on the XIAP regulation by miR-200b-3p, but it is well known that one miRNA has multiple targets. Consequently, we next investigated whether the adenosine methylation of miR-200b-3p could abrogate its translational repressor function toward putative protein targets other than XIAP. Among the putative protein targets of miR-200b-3p (according to the miRTarBase website^24^), we focused our study on two other apoptotic players (Bcl-2 [B cell lymphoma 2, UniProt: P10415] and caspase-2 [cysteine-dependent aspartate-directed proteases 2, UniProt: P42575]), two epigenetic players (EZH2 [enhancer of zeste homolog 2, UniProt: Q15910] and DNMT1 [DNA (cytosine-5)-methyltransferase 1, UniProt: P26358]), and the negative immune checkpoint PD-L1 (programmed cell death 1 ligand 1, UniProt: Q2NZQ7). Our data indicated that the presence of m6A in miRNA-200b-3p also abrogated the translational repressor function of miR-200b-3p toward Bcl-2 and PD-L1 (Figure 4A).

Finally, we investigated whether the ability of m6A-miR-200b-3p to induce cell death was specific to U87 cells. For this purpose, cancerous cell lines representative of several cancers were transfected with m6A-miR-200b-3p (U251 and T98G for glioblastoma, A549 and H1975 for lung, MCF7 and T47D for breast, OE21 for esophagus, OV90 and SKOV3 for ovaries). Four non-cancerous cell lines were also included in our study. Four hours after cells transfection, we noted that all cells were transfected with a similar quantity of m6A-miR-200b-3p since the range of increase of miR-200b-3p expression was homogeneous (10- to 13-fold induction) (Figure 4B). Then, we noted that cell death occurred in cells having the ability to adenosine-demethylate miR-200b-3b, i.e., in U251, A549, T47D, and SKOV3 cells (Figure 4B). The absence of cell death in other cell lines and particularly in non-cancerous cell lines was explained by the inability of these cells to adenosine-methylate miR-200b-3b (with the m6A enrichment-transfected/control being equal to 1) (Figure 4B).

Taken together, all of the latter results are consistent with the fact that m6A-miR-200b-3p appears as a promising tool in anti-GBM therapy.

## Discussion

Recent investigations concerning the description of the molecular mechanisms of base modifications of miRNAs have provided meaningful progress in the understanding of regulation of the biogenesis and functionality of miRNAs. Thus, after the studies of Alarcón et al.,^5^ Berulava et al.,^6^ and Konno et al.,^7^ our study reports the presence of m6A in miRNAs via the realization of RNA IP with an anti-m6A antibody followed by qRT-PCR. Beyond these earlier studies, our investigation provides several innovative points.

First, our work indicates that the adenosine methylation of miR-200b-3p abrogates its translational repressor function toward its putative targets such as XIAP, Bcl-2, and PD-L1. The works by Alarcón et al.^5^ and Berulava et al.^5^ reported the existence of two different consensus sequences for the m6A methylation in pri-miRNAs (UGAC) and in mature miRNAs (ADRA).^5^^,^^6^ Interestingly, we noted that the miRNA-200b-3p sequence contains a sequence matching one of the consensus sequences (Table S2). We also noted that the miR-200b-3p sequence contains a sequence matching the consensus sequence binding by METTL3/WTAP as defined by Ping et al.^12^ (Table S2). From a certain perspective, this last point can also constitute an argument supporting the role of METTL3 in the adenosine methylation of miRNAs.

The work of Berulava et al.^6^ indicated that FTO plays a crucial role in the demethylation of miRNAs. Our data complete this by indicating that the presence of αKG also acts as a non-negligible player in the demethylation of miRNAs.

In addition to these two initial reports, our study shows that the presence of m6A acts as an inhibitor of the post-transcriptional repressor function of miRNAs. Mechanistically, our data indicate that the presence of m6A limits the formation of the miRNA/mRNA duplex. Our study is also distinguished from the first two studies by its clinical translational study effort using a cohort of cancer patients. Indeed, our study is the first to mention that the level of N6-adenosine methylation of a miRNA (in association with the expression level of this miRNA) acts as a biomarker characterizing GBM patients with a poor survival. Our study is also distinct from the one recently published by Konno et al.^7^ in which they considered the adenosine methylation of miRNA as a tool to distinguish early pancreatic cancer patients from healthy controls with an extremely high sensitivity and specificity; however, in our article the adenosine methylation of miR-200b-3p is associated with a prognosis value of response for GBM patients and could have a therapeutic function.

The work of Berulava et al.^6^ and that of Yuan et al.^25^ introduced a debate about the impact of the adenosine methylation of miRNAs on their stability. Our data focusing on miR-200b-3p seems to indicate that the adenosine methylation of this miRNA does not affect its expression. Indeed, the modulation of its adenosine methylation level via siRNA directed against FTO and METTL3 or via chemical components does not affect its expression. However, because this finding was obtained on one miRNA, it is not possible to generalize a rule about the impact of adenosine methylation on miRNA stability.

By observing that the adenosine-methylated miR-200b-3p was not recruited to the RISC complex, our data reinforce the idea that the adenosine methylation of miRNA appears as a molecular mechanism governing the miRNA functionality via the regulation of the duplex formation between miRNA and mRNA. More generally, our data support the idea that nucleotide modification occurring in miRNA or in 3′ UTR-mRNA alters the formation of the miRNA/3′ UTR-mRNA duplex, such as reported by Lockhart et al.^26^

By reporting that m6A methylation of miRNAs could act as a biomarker characterizing GBM patients with a poor survival, our data open the idea that the molecular actor writing this epitranscriptomic signature (METTL3 according to our data) could be used as a target for the development of epidrugs. Indeed, this point of view is already discussed since METTL3 promotes oncogene translation.^27^

During the last decade, miRNA mimics and molecules targeting miRNAs (anti-miRNAs) have shown promising results in preclinical development.^28^^,^^29^ Four arguments strongly support the idea that the adenosine-methylated form of miR-200b-3p could be used as a promising therapeutic tool. First, m6A-miR-200b-3p is apoptogenic by itself via the repression of XIAP, an anti-apoptotic protein. Second, our data indicate that m6A-miR-200b-3p promotes cell death in cancerous cells such as U87 (but also in other cancer cell lines) and not in non-cancerous cells such as neurons, PBMCs, astrocytes, and hepatocytes. Third, our *in vivo* data indicate that m6A-miR-200b-3p has an anti-tumor growth effect in an *in vivo* model of GBM. Fourth, our *in vivo* data also indicate that the m6A-miR-200b-3p/TMZ combination permits to limit the dose of TMZ since the m6A-miR-200b-3p/TMZ (25 mg/kg) combination has the same anti-tumor growth effect than does the use of the TMZ (50 mg/kg) treatment. Thus, all of these arguments define the adenosine-methylated form of miR-200b-3p as the prodrug form of this miRNA. More interestingly, our data indicate that its conversion under an active form occurs in cancer cells but not in non-cancerous cells. This observation is highly promising since it can be translated such as the fact that only cancerous cells have the “tools” (FTO and αKG) to activate the prodrug form of miR-200b-3p. Thus, the adenosine-methylated form of miRNAs could be considered as such a manner to limit the effect of off-targets of miRNA therapy associated with the relative lack of addressing miRNA-based therapy against cancer cells.^30^ Our data also introduce the idea that the presence of IDH1 mutations could be considered as a biomarker excluding the use of the adenosine-methylated form of miRNAs since cells presenting IDH1 mutations have a low level of αKG. Concretely, the first reading of this idea might exclude the use of m6A-miR-200b-3p treatment in less than 10% of primary GBM and in 6%–10% of *de novo* acute myeloid leukemia (AML), for example.^31^^,^^32^ However, this point is applicable when the m6A-miR-200b-3p treatment is envisioned as a single treatment since its combination with BAY1436032 (a pan mutant IDH1 inhibitor^33^) restored its ability to promote cell death (Figure S6).

In conclusion, our study opens a new area in the understanding of epigenetic modifications concerning miRNA and in the development of innovative epidrugs. Indeed, for several years chemical modifications of RNAs (i.e., epitranscriptomic) have been defined as central players in the control of messenger and ncRNA activity.^34^ Our data reinforce this idea by showing that the adenosine methylation of miRNAs abrogates their post-transcriptional repressive function. By initiating the idea that adenosine-methylated miRNA could be used as a prodrug, our work provides the base for the development of a new pathway of anti-cancer therapeutic strategies targeting miRNA. Thus, in future years, the understanding of the mechanisms involved in the epigenetic regulation of miRNA could improve patient stratification and the development of successful miRNA-based therapeutic strategies.

## Materials and Methods

### miRNA Extraction

miRNA extractions were performed using the NucleoSpin miRNA kit (Macherey Nagel, France) according to the manufacturer’s instructions.

### miRNA and siRNA Transfection

Briefly, 6 × 10^5^ cells were seeded in each well of four-well plates. Transfection was performed using HiPerfect transfection reagents (QIAGEN, France) and 10 ng of miRNA (QIAGEN, France) or 10 nm of Silencer siRNA (Thermo Fisher Scientific, France), according to the manufacturers’ recommendations. For siRNA controls, transfection control (HiPerfect transfection reagent only) and a negative control (silencer negative control 1 siRNA) were used. For miRNA controls, transfection control (HiPerfect transfection reagent only) and an oligonucleotide (miScript inhibitor negative control; QIAGEN, France) were used.

### Acellular METTL3 Methylation Assay

METTL3-including complexes were immunoprecipitated from cellular lysate obtained after sonication and the use of 3-[(3-cholamidopropyl)dimethylammonio]-1-propanesulfonate (CHAPS) buffer (40 mM HEPES [pH 7.4], 120 mM NaCl, 1% CHAPS, and 1 mM EDTA, supplemented with protease and phosphatase inhibitors). IPs were performed using the Catch and Release v2.0 reversible IP system (Merck, France) and anti-METTL3 (Abcam, France). Immunoglobulin G (IgG) (Abcam, France) was used as a control. Elutions from IP were performed using the non-denaturing elution buffer according to the manufacturer’s instructions. Then, 30 μL of elution was used in the METTL3 enzymatic assay. The METTL3 enzymatic assay was conducted in reaction buffer (20 mM Tris [pH 7.5], 1 mM DTT, 0.01% Triton X-100, and 40 U/100 mL of RNaseOUT buffer). The reaction mixture contained unmethylated mimic miR-200b-3p with biotin tag and S-adenosyl methionine (SAM). Enzymatic assay reactions were incubated overnight at room temperature on a shaker. After streptavidin isolation, the presence of N6-adenosine methylation was determined by a dot blot. Dots were then incubated with anti-m6A and anti-adenosine (as a loading control) antibodies overnight. For signal detection, secondary horseradish peroxidase (HRP) antibodies were used and signal was detected on ChemiDoc MP (Bio-Rad, France).

### RIP for miRNA

For IP of RNA, two rounds using 5 μg of anti-m6A antibody (Abcam, France) and 5 μg of small RNA were performed. The reaction was carried out using a Dynabeads protein G IP kit with some modifications (Thermo Fisher Scientific, France) such as described by Berulava et al.^6^ As a control, IP was performed using IgG (Abcam, France) instead of anti-m6A antibody. miRNAs obtained from m6A IP were reverse transcribed using miRScript II RT kit (QIAGEN, France) and analyzed using the miScript miRNA PCR array human cancer pathway kit (QIAGEN, France) according to the manufacturers’ instructions. Fold enrichment was next calculated using Ct value obtained from qRT-PCR performed with input miR, IP-IgG, and IP-m6A, and the 2^−ΔΔCt^ formula.

### CLIP

CLIP assays were performed using a RiboCluster Profiler RIP assay (CliniScience, France) from 10 million per sample of UV-crosslinked cells (150 mJ/cm^2^ of UVA [365 nm]) according to the manufacturer’s instructions. IP assays were performed in the presence of 15 μg of anti-GW182 (no. RN033P, CliniScience, France) and anti-TNRC6B (no. 9913, Merck Millipore, France) overnight at 4°C.

### qPCR of miRNA

For miRNA expression analysis and detection from products of RIP performed with anti-m6A antibody, RNA was reverse transcribed using a miScript II RT kit and analyzed by qPCR with the miScript SYBR Green PCR kit using the specific hsa-miR miScript primer assays (QIAGEN, France) according to the manufacturer’s instructions.

### ELISA

Proteins extracts were obtained by using radioimmunoprecipitation assay (RIPA) lysis and extraction buffer (Thermo Scientific, France) in accordance with the manufacturer’s instructions. An XIAP (human) cell-based ELISA kit (Abnova, Taiwan), αKG assay kit (no. ab83431, Abcam, France), human FTO ELISA kit (no. 68ELH-FTO, Tebu-Bio, France), METTL3 ELISA kit (no. MBS9326769, My BioSource, USA), CST-PathScan total Ezh2 sandwich ELISA kit (Ozyme, France), EpiQuik Dnmt1 assay kit (Euromedex/EpiGentek, France), human Bcl-2 ELISA kit (Abcam, France), caspase-2 ELISA kit (Tebu-Bio, France), and PathScan total PD-L1 sandwich ELISA kit (Ozyme, France) were performed according to the manufacturers’ instructions.

### Tumor Xenografts in Nude Mice

Cells were harvested by trypsinization, washed, and resuspended in saline buffer. Cell suspensions were injected subcutaneously (s.c.) into the flank of 7- to 8-week-old mice (Janvier, France) in 100 μL of sterile PBS. Tumor volume based on caliper measurements was calculated using the modified ellipsoidal formula as follows: Tumor volume = ½(length × width2).

The experimental procedures using animals were in accordance with the guidelines of Institutional Animal Care and the French National Committee of Ethics. In addition, all experiments were conducted according to the Regulations for Animal Experimentation at the “Plateforme Animalerie” of Institut de Recherche en Santé de l’Université de Nantes (IRS-UN) and approved by the French National Committee of Ethics (agreement no. B44278).

### Cell Lines

U87, U87^IDH1mut^, RN33b, and A549 cells were obtained from the American Type Culture Collection (ATCC, Molsheim, France). HASTR040/astrocytes were obtained from Clonexpress (Gaithersburg, MD, USA). OE21 cells were obtained from Sigma (France). HEP10 cells were obtained from Thermo Fisher Scientific (France). MCF7 and T47D cells were provided by the Dr. P. Juin’s laboratory. SKOV3 cells were provided by the Dr. E. Scottet’s laboratory. OV90 cells were provided by the Dr. R. Spisek’s laboratory.

### Statistical Analysis

All experiments were done at least in triplicates. Significance of the differences in means were calculated using a Student’s t test while correlations were determined using Pearson’s test. Survival curves were plotted according to the Kaplan-Meier method and compared with a log-rank test.

### Ethics Approval and Consent to Participate

Patient material as well as records (diagnosis, Karnofsky performance status [KPS], age, sex, date of death) was used with confidentiality according to French laws and recommendations of the French National Committee of Ethics. In addition, patient material and experiments using this material are conducted according to the regulations of “the Réseau des Tumorothèques du Cancéropôle Grand-Ouest” and more particularly with the regulations of “Réseau Gliome.” All patients provided informed consent in accordance with the Declaration of Helsinki.

## Author Contributions

P.-F.C. designed and coordinated the project. J.B., A.A.S., A.N., G.B.-C., and P.-F.C. performed all experiments. F.M.V., A.A.S., B.O., D.H., and P.F.C. interpreted and discussed the data. P.-F.C. wrote the first version of the manuscript, and all authors reviewed and approved it.

## Conflicts of Interest

The authors declare no competing interests.

## Supplemental Information

Document S1. Figures S1–S6 and Tables S1 and S2

Document S2. Article plus Supplemental Information
