# Supplementary material for: N6-Adenosine Methylation of miRNA-200b-3p Influences Its Functionality and Is a Theranostic Tool
Source: Mol Ther Nucleic Acids. 2020 Aug 14;22:72–83. doi: 10.1016/j.omtn.2020.08.010 (PMC7490450; doi:10.1016/j.omtn.2020.08.010)
Supplement: Document S1. Figures S1–S6 and Tables S1 and S2 [file mmc1.pdf]

OMTN, Volume *22*

Supplemental Information

N6-Adenosine Methylation of miRNA-200b-3p Influences Its Functionality and Is a Theranostic Tool

Joséphine Briand, Aurélien A. Sérandour, Arulraj Nadaradjane, Gwenola Bougras-Cartron, Dominique Heymann, Benjamin Ory, François M. Vallette, and Pierre-François Cartron

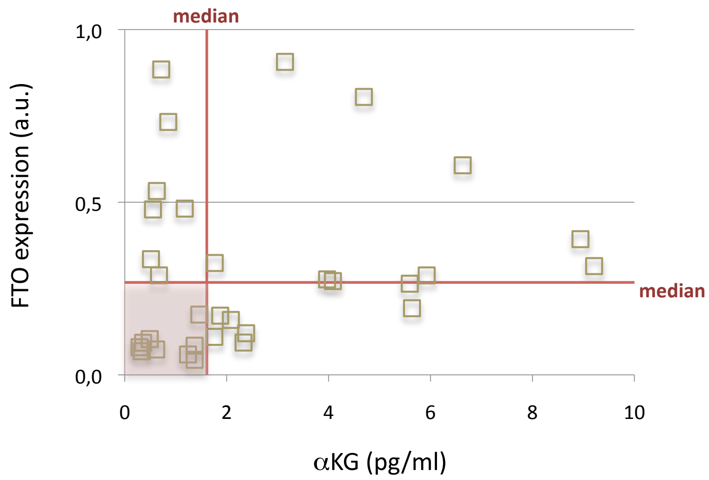

Figure S1. **Patients stratification based on the FTO and αKG levels seen in their GBM.**

By using the median value of the two considered parameters, we identified a sub-population of patients harboring a low level of FTO and a low level of αKG (in red area). Each square symbolizes a GBM sample (n=32). ELISA were used to quantified the FTO and αKG expression.

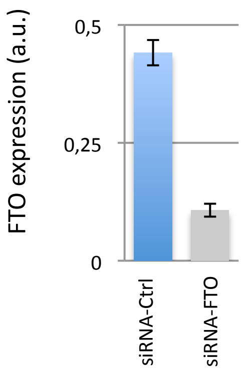

Figure S2. **Impact of the siRNA-mediated FTO invalidation on the adenosine methylation percentage of miR-200b-3p (miR-200b-3p%^{m6A}) in U87 cells.**
Human FTO ELISA kit was used to estimate the impact on FTO expression of cell treatment with si-RNA control *Silencer®* Negative Control #1 siRNA and si-RNA directed against FTO *Silencer®* FTO siRNA (ThermoFisher Scientific) . Experiments were performed according to the manufacturer' instructions.

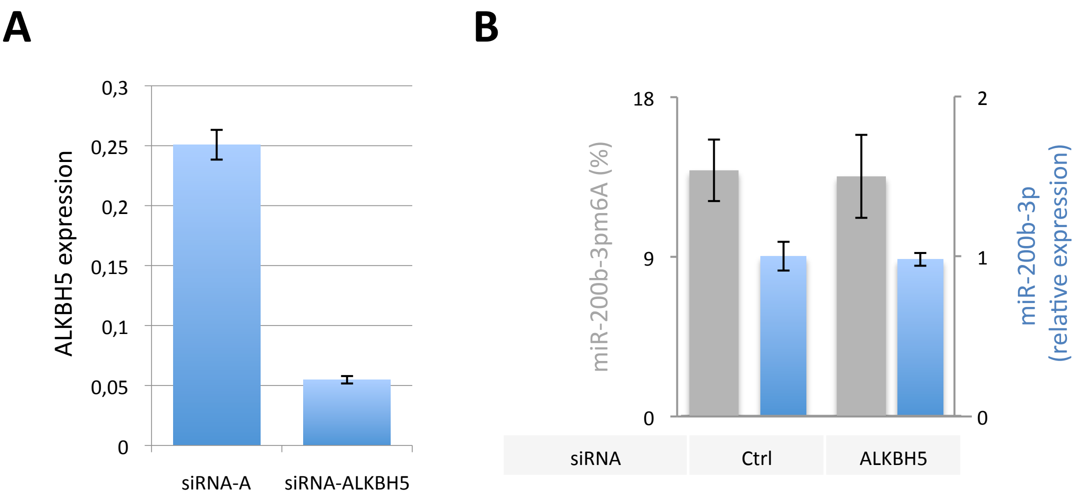

Figure S3. Impact of the ALKBH5 invalidation the adenosine methylation percentage of miR-200b-3p (miR-200b-3p%^{m6A})) in U87 cells.

A. Human ALKBH5 ELISA kit was used to estimate the impact on ALKBH5 expression of cell treatment with si-RNA control *Silencer*® Negative Control #1 siRNA (Ctrl) and siRNA directed against ALKBH5 *Silencer*® ALKBH5 siRNA (ThermoFisher Scientific, France) . Experiments were performed according to the manufacturer' instructions.

B. miR-200b-3p%^{m6A} was calculated trough the realization of miRIP^{6mA}-qPCR as previously described.

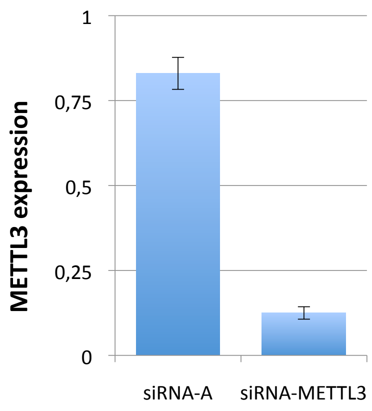

Figure S4. **siRNA-induced invalidation of METTL3) in U87 cells.**

Human METLL3 ELISA kit was used to estimate the impact on METLL3 expression of cell treatment with si-RNA control *Silencer®* Negative Control #1 siRNA and si-RNA directed against METLL3 *Silencer®* METLL3siRNA (ThermoFisher Scientific, France) . Experiments were performed according to the manufacturer' instructions.

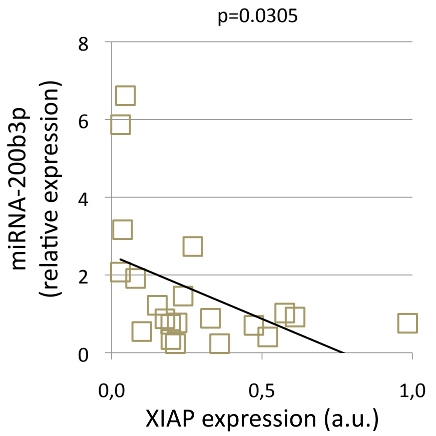

Figure S5. **Correlation, in tumors having a miR200b-3p$^{\%6mA}$<10% (n=22), between the relative expression level of miRNA-200b-3p (qPCR experiments) and XIAP expression (ELISA).**

p-value is estimated from the Pearson Correlation Coefficient Calculator (r=-0.4618).

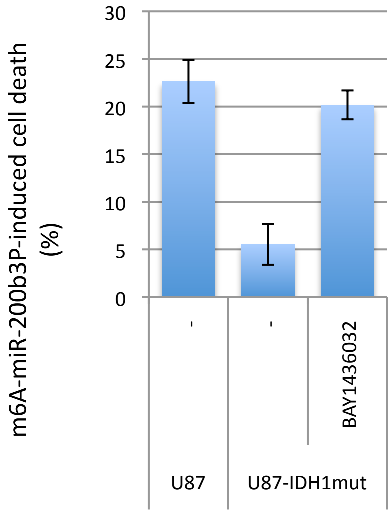

Figure S6. **IDH1 inhibitor (50µM, BAY1436032) restores cell death in U87^{IDH1mut} cells.**

The LDH-Cytotoxicity Assay Kit (Abcam, France) is used to estimate the cell death 24h after the m6A-miR-200b-3b and/or BAY1436032 incubations (IDH inhibitor).

Characteristics		Patients (n=32)
Age (years)<br>Median (range)		59 (43;76)
Sex		
	Male (%)	19 (59)
	Female (%)	13 (41)
Karnofsky performance score		
	60-80 (%)	22 (69)
	90-100 (%)	10 (31)
Survival time (months)		
Median (range)		16(6;42)
Extent of surgery (%)		
	Biopsy	0
	Partial resection	0
	Complete resection	32 (100)

Table S1. **Clinical characteristics of GBM's patients included in our study.**

	Reference	-3	-2	-1	0	+1	+2	Homology (%)
**m6A in mRNA and lncRNA**	Berulava et al. PMID: 25723394		G/A	G/A	**A**	C	A/U/G	60
**Discriminated motif for m6A in miRNA**	Berulava et al. PMID: 25723394	A	A/G/U	G/A	**A**			100
**METTL3/WTAP motif**	Ping et al. PMID: 24407421	A/U	G/U	G/U	**A**	C/U	U/G	100
**FIRE motif discovery analysis of the METTL3 HITS-CLIP binding sites in pri-miRNA**	Alarcón et al. PMID: 25799998		U	G	**A**	C		75
**miRNA-200b-3p**	miRBase	**A**	**U**	**G**	**A**	**U**	**G**	

Table S2. **Alignment of the miRNA-200b-3p sequence with consensus sequences of interest.**
